# Intravascular haemolysis with haemoglobinuria in a splenectomized patient with severe *Plasmodium knowlesi* malaria

**DOI:** 10.1186/s12936-016-1514-0

**Published:** 2016-09-09

**Authors:** Bridget E. Barber, Matthew J. Grigg, Timothy William, Tsin W. Yeo, Nicholas M. Anstey

**Affiliations:** 1 Menzies School of Health Research and Charles Darwin University, PO Box 41096, Casuarina, NT 0810 Australia; 2 Infectious Diseases Society Sabah-Menzies School of Health Research Clinical Research Unit, 88586 Kota Kinabalu, Sabah Malaysia; 3 Queen Elizabeth Hospital Clinical Research Centre, 88586 Kota Kinabalu, Sabah Malaysia; 4 Jesselton Medical Centre, 88300 Kota Kinabalu, Sabah Malaysia; 5 Lee Kong Chian School of Medicine, Nanyang Technological University, Singapore, 639798 Singapore

**Keywords:** *Plasmodium knowlesi*, Malaria, Haemolysis, Splenectomy, Blackwater fever, Artesunate

## Abstract

**Background:**

Haemoglobinuria is an uncommon complication of severe malaria, reflecting acute intravascular haemolysis and potentially leading to acute kidney injury. It can occur early in the course of infection as a consequence of a high parasite burden, or may occur following commencement of anti-malarial treatment. Treatment with quinine has been described as a risk factor; however the syndrome may also occur following treatment with intravenous artesunate. In Malaysia, *Plasmodium knowlesi* is the most common cause of severe malaria, often associated with high parasitaemia. Asplenic patients may be at additional increased risk of intravascular haemolysis.

**Case presentation:**

A 61 years old asplenic man was admitted to a tertiary referral hospital in Sabah, Malaysia, with severe knowlesi malaria characterized by hyperparasitaemia (7.9 %), jaundice, respiratory distress, metabolic acidosis, and acute kidney injury. He was commenced on intravenous artesunate, but1 day later developed haemoglobinuria, associated with a 22 % reduction in admission haemoglobin. Additional investigations, including a cell-free haemoglobin of 10.2 × 10^5^ ng/mL and an undetectable haptoglobin, confirmed intravascular haemolysis. The patient continued on intravenous artesunate for a total of 48 h prior to substitution with artemether–lumefantrine, and made a good recovery with resolution of his haemoglobinuria and improvement of his kidney function by day 3.

**Conclusions:**

An asplenic patient with hyperparasitaemic severe knowlesi malaria developed haemoglobinuria after treatment with intravenous artesunate. There are plausible mechanisms for increased haemolysis with hyperparasitaemia, and following both splenectomy and artesunate. Although in this case the patient made a rapid recovery, knowlesi malaria patients with this unusual complication should be closely monitored for potential deterioration.

## Background

Haemoglobinuria reflects acute intravascular haemolysis, and can occur as a complication of severe malaria, potentially leading to acute kidney injury. While it can occur early in the course of infection as a direct consequence of a high parasite burden and rupture of parasitized and unparasitized cells, it may also occur as a complication of anti-malarial treatment. In previous reports, haemoglobinuria occurring in patients with malaria has been referred to as blackwater fever. While definitions vary, the term was historically used to describe a clinical syndrome of haemoglobinuria, fever and jaundice that typically involved non- or partially immune European expatriates who had been residing in malaria-endemic areas and who had received, often intermittently, treatment with quinine. More recent reports however, have described blackwater fever or haemoglobinuria in Southeast Asian adults [1] and in African children [2–5], including in those treated with artesunate [1, 6–8]. The syndrome is most commonly associated with falciparum malaria, although has also been reported with *Plasmodium vivax* [9] and with mixed species infections [1, 3], and in severe knowlesi malaria [10, 11].

The comparative risk of intravascular haemolysis in splenectomized patients with malaria has not been evaluated. Splenectomized patients are thought to be at increased risk of developing complications from malaria, and in patients with thalassaemia, asplenic patients experience more severe intravascular haemolysis [12]. *Plasmodium knowlesi* is the most common cause of malaria in Malaysia and is associated with high parasitaemia infections [13]. In rhesus macaques with high parasitaemia *P. knowlesi* infections, haemoglobinuria was commonly a pre-terminal event [14, 15]. This report describes a case of haemoglobinuria that occurred in a splenectomized patient with severe knowlesi malaria, following treatment with intravenous artesunate.

## Case presentation

A 61 years old farmer presented to Kudat District Hospital in northeastern Sabah with a 3-day history of fever, rigours, cough, headache, arthralgia, and myalgia. He lived in a village near Kudat town and had recently travelled to Banggi Island off the coast of Sabah, at the time highly endemic for malaria, where he had stayed overnight in forested areas, and had seen monkeys. His past history was significant for having undergone a splenectomy 5 years previously following a motor vehicle accident, hypertension, and self-reported malaria 10 years previously. His medications included life-long prophylactic penicillin and perindopril. He denied having taken any anti-malarial medications prior to presentation.

**Fig. 1:**
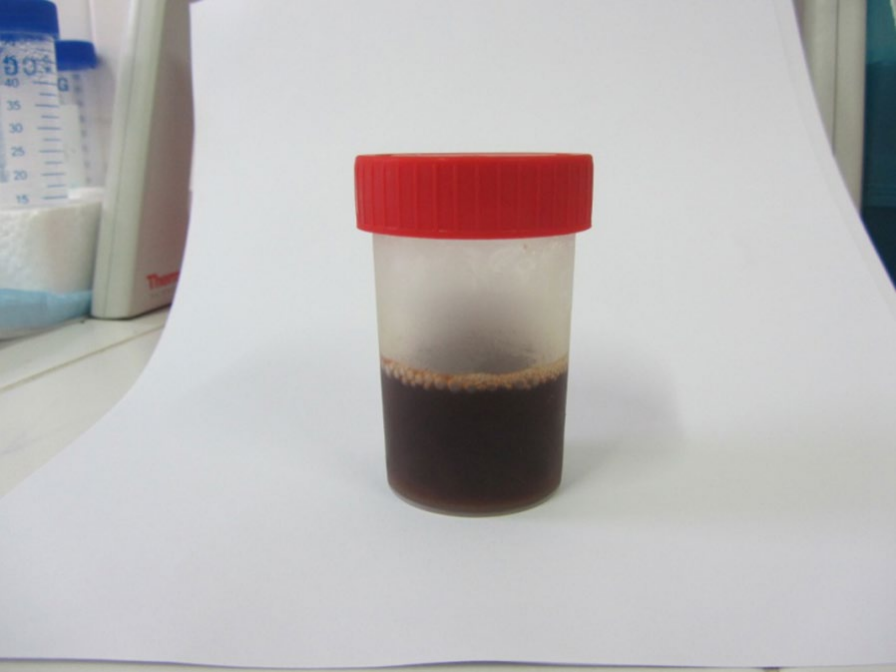
Urine sample on day 1, after completion of two doses of intravenous artesunate

On examination his temperature was 38.9 °C, heart rate 93 beats per minute, blood pressure 114/79 mm Hg, respiratory rate 36 breaths/minute and oxygen saturation 88 % on room air. He was notably jaundiced and had a scar on his abdomen, but examination was otherwise unremarkable. His urine was of normal colour. Blood film was reported as *P. knowlesi*, with a parasite count of 7.9 %. His haemoglobin was 15.2 g/dL, white cell count 8.7 × 10^3^/μL, platelets 24 × 10^3^/μL, and creatinine 145 µmol/L (Table 1). He was commenced on intravenous artesunate in addition to ceftriaxone, and transferred to a tertiary referral hospital. An arterial blood gas taken the following morning on 35 % oxygen via a Venturi mask revealed metabolic acidosis with a pH of 7.31 and bicarbonate of 11 mmol/L. His chest X-ray was unremarkable. One day later, after two doses of intravenous artesunate given on admission and at 12 h, he was noted to have “coca-cola” coloured urine (Fig. 1), with urinalysis positive for haemoglobin with no intact red blood cells. Additional blood investigations revealed a bilirubin of 181 µmol/L and elevated liver transaminases (Table 1). Glucose-6 phosphate dehydrogenase (G6PD) activity was normal, thalassemia screen was negative, and dengue NS1 antigen was negative. Testing for leptospirosis was not performed. The patient received two further doses of artesunate (at 24 and 48 h) before changing to artemether–lumefantrine. He made a good clinical recovery, with improvement of his oxygen saturation, jaundice, thrombocytopaenia, and renal function (creatinine 86 µmol/L on day 3; Table 1). By day 3 he was afebrile with no malaria parasites seen on blood film, and his haemoglobinuria had largely resolved. PCR confirmed *P. knowlesi* mono-infection. No pathogens were isolated from blood cultures taken after commencement of antibiotics. The patient received ceftriaxone for a total of 7 days.

**Table 1:** Laboratory values

	Day 0	Day 1	Day 2	Day 3	Day 4
Parasite count (parasites/µL)	431,000^a^	56,000	14,880	Negative	Negative
Haemoglobin (g/dL)	15.2	13.3	11.8	12.5	12.2
Haematocrit (%)	43.4	39.5	33.4	35.5	35.3
White blood cells (×10^3^/µL)	8.7	10.9	10.7	11.5	11.9
Platelets (×10^3^/µL)	24	28	29	52	104
Creatinine (µmol/L)	145	112	92	86	
Total bilirubin (µmol/L)		181	178	57	
Direct bilirubin (µmol/L)		110	115	33	
Alanine aminotransferase (IU/L)		90	62	55	
Aspartate aminotransferase (IU/L)		146	160	71	
Bicarbonate (mmol/L)		11	23.1	23.9	
Lactate (mmol/L)		1.89		1.74	1.30
Glucose (mmol/L)	8.0	12.1	10.8	9.3	
Cell free haemoglobin (ng/mL)		10.2 × 10^5^		0.3 × 10^5^	
Haptoglobin (µg/mL)		<0.01			

^a^ Parasitaemia as reported by expert research microscopist. A total of 30 ring stage parasites, 35 trophozoites and 14 schizonts per 1000 RBCs were seen, accounting for a parasitaemia of 7.9 %, or 431,000 parasites/μL

As the patient was enrolled in a prospective pathophysiology study, venous blood was collected (14.5 h after commencement of intravenous artesunate) in lithium heparin and citrate tubes and centrifuged within 30 min, with plasma stored at −80 °C. Cell-free haemoglobin and haptoglobin were measured by enzyme-linked immunosorbent assay (ELISA), revealing markedly elevated cell-free haemoglobin (10.2 × 10^5^ ng/mL) and undetectable haptoglobin.

## Discussion

This report describes a case of knowlesi malaria in a splenectomized patient, with WHO-defined criteria for severe disease, including hyperparasitaemia, respiratory distress, metabolic acidosis, and jaundice [16], who developed haemoglobinuria (sometimes referred to as blackwater fever) following treatment with artesunate. This is the second reported case of haemoglobinuria in a patient with severe knowlesi malaria treated with artesunate [10], and, given the potential for association with acute kidney injury, highlights the importance of monitoring for this complication in such patients, particularly in those who are splenectomized.

The haemoglobinuria in this patient developed on the day following admission, and laboratory investigations, including a 22 % drop in haemoglobin, undetectable haptoglobin and massively elevated cell-free haemoglobin, all confirmed intravascular haemolysis. The cause of this haemolysis is likely multifactorial. In the setting of hyperparasitaemia, rupture of parasitized red blood cells (RBCs) alone can be expected to cause substantial haemolysis; however the degree of anaemia in this case implies additional loss of unparasitized RBCs. In falciparum malaria, factors that may contribute to lysis of non-parasitized RBCs include the direct effects of parasite products [17], inflammatory cytokines [18], complement activation [17, 19], and membrane lipid peroxidation [18]. However, in the current case the haemoglobinuria occurred only after the patient received two doses of intravenous artesunate, and it is therefore possible that artesunate may have contributed to the haemolysis.

While haemoglobinuria is well documented as a complication of quinine and other arylamino alcohol drugs, the link with artesunate is less well described. However, recent studies suggest that rates may be similar to those seen with quinine. In the AQUAMAT study involving African children with severe falciparum malaria, blackwater fever was reported in 18/2597 (0.7 %) patients following treatment with intravenous artesunate compared to 30/2591 (1.2 %) following treatment with intravenous quinine [6]. Blackwater fever was more common in the SEAQUAMAT study involving Southeast Asian adults and children with severe falciparum malaria, being reported in 49/730 (7 %) and 33/731 (5 %) following treatment with artesunate and quinine, respectively [8]. In a smaller study, haemoglobinuria occurred in 3/76 (3.9 %) Ugandan children receiving intravenous artesunate for severe falciparum malaria [7].

In addition to these reports of haemoglobinuria occurring following treatment with artesunate, there are numerous reports of artesunate-associated haemolytic anaemia occurring without haemoglobinuria [20–22]. Jaureguiberry et al. described three patterns of haemolytic anaemia occurring in patients with falciparum malaria treated with artesunate: (1) a ‘rising’ pattern, in which the nadir haemoglobin and peak of haemolysis occur before day 8; (2) a delayed pattern (post-artesunate delayed haemolysis; PADH), defined by a >10 % drop in haemoglobin or a >10 % rise in lactate dehydrogenase (LDH) occurring after day 8; and, (3) a ‘persistent pattern’, in which anaemia and haemolysis occur before and after day 8 [22]. In a study of 60 non-transfused travellers with falciparum malaria treated with intravenous artesunate, these patterns of post-artesunate haemolytic anaemia occurred in 32, 17 and 22 % of patients, respectively, with the rising pattern (as occurred in the current case) associated with a mean 21 % decline in haemoglobin and marked haemolysis until day 4 [22]. The association between these patterns of artesunate-associated haemolytic anaemia and the occurrence of haemoglobinuria however remains uncertain.

The mechanisms of acute artesunate-related haemolysis are unclear. One mechanism contributing to PADH is the splenic removal of parasites from RBCs, with these ‘pitted’ once-infected RBCs then returned to the circulation but with reduced lifespan [22]. This mechanism however is less likely to explain acute haemolysis. In addition, the degree of anaemia in this case, and in previous reports of PADH [23], suggests that artesunate may also contribute to haemolysis of non-parasitized RBCs. Artesunate contains a highly active endoperoxide bridge that, cleaved in the presence of haem, generates reactive oxygen species and other free radicals [24–27]; it is possible that this oxidative stress may contribute to haemolysis of RBCs. Artesunate has also been shown to induce phosphatidylserine (PS) translocation at the RBC membrane [27]. PS is a membrane phospholipid which is normally located on the internal leaflet of the lipid bilayer, however may become exposed when cells undergo oxidative stress, or during parasite maturation [28, 29]. PS-RBCs have been shown to play a role in inflammation [30], coagulation [31], platelet activation [32], and adhesion to vascular endothelial cells [33], and may increase susceptibility to haemolysis [12].

In this case, ceftriaxone was another possible cause of drug-induced haemolysis; haemolysis attributed to ceftriaxone has been previously reported in a patient with severe falciparum malaria [34]. However, in the current case, the patient’s haemoglobinuria resolved despite continuation of ceftriaxone for a total of 7 days, making this unlikely. G6PD deficiency is a known risk factor for blackwater fever [1]; however, was not present in this case.

In the current case, the lack of a spleen likely contributed to the severity of intravascular haemolysis, and may have increased the risk of haemoglobinuria. In patients with haemoglobin E/β-thalassaemia disease, splenectomy has been shown to be associated with increased intravascular haemolysis, possibly due to an absence of splenic filtering of aged and/or defective RBCs [12]. In addition, PS-RBCs have been shown to be increased in splenectomized individuals [30, 31]. Blackwater fever in a splenectomized patient with falciparum malaria has been reported [35]. However, whether risk of haemolysis is increased in splenectomized patients with malaria has not been evaluated.

This is the sixth report of knowlesi malaria to occur in a splenectomized patient. Previous reports include two cases of uncomplicated malaria [10] and three cases of severe malaria [10, 36, 37], one of which occurred in a patient with β-thalassaemia and was transfusion-acquired [37]. Of the four severe cases (including the current case), all had jaundice, respiratory distress and metabolic acidosis, with two also complicated by acute kidney injury requiring dialysis [10, 36]. Not unexpectedly, in two cases parasite clearance was markedly delayed [10, 36]. In the two uncomplicated cases there was an absence of thrombocytopaenia [10], with this finding being notable due to the near-universal finding of thrombocytopaenia with knowlesi malaria in patients with intact spleens [10, 13]. An increase in platelet counts has also been reported in thalassaemic patients who are splenectomized; while the mechanisms are unclear, an increase in PS-RBCs may be contributory [12].

In this case, although stage 1 AKI [by Kidney Disease Improving Global Outcomes (KDIGO)] criteria [38] was present on admission, the patient made a rapid recovery with anti-malarial treatment. In previous series, AKI has been a common complication of haemoglobinuria/blackwater fever. In Vietnamese adults 42 % of cases had acute renal failure [1], while renal failure was seen in 16 % of Congolese children with blackwater fever [3]. In European expatriates, renal failure occurred in 70 % of cases with blackwater fever [39]. Haemoglobinuria has also been associated with AKI in other diseases, including babesiosis [40], paroxysmal nocturnal haemoglobinuria [41–43], and post-cardiopulmonary bypass [44]. While the mechanisms of haemolysis-induced AKI remain uncertain, direct tubular cell injury from free haem likely contributes [23, 45]. In addition, free haem has been shown to cause oxidative damage by lipid peroxidation, leading to renal injury through vasoconstriction [46, 47]. Finally, cell-free haemoglobin is a scavenger of nitric oxide (NO) and in severe falciparum malaria has been shown to be associated with reduced NO-dependent endothelial function and impaired tissue perfusion [48], possibly also contributing to renal injury.

## Conclusions

This report describes a case of a splenectomized patient with severe knowlesi malaria who developed haemoglobinuria following commencement of treatment with artesunate. The AKI in this case was not severe, antedated the artesunate and the patient made a rapid recovery despite continuation of artemisinins. Artesunate reduces mortality in severe falciparum malaria [6, 8] and is associated with lower case-fatality than quinine in severe knowlesi malaria [10, 11, 49], making it the clear treatment of choice for severe disease in knowlesi malaria [16]. Clinicians should however be aware of the possibility of this rare complication in knowlesi malaria with high parasitaemia so that patients can be adequately monitored for potential deterioration.

## Abbreviations

AKI: acute kidney injury
ELISA: enzyme-linked immunosorbent assay
G6PD: glucose-6 phosphate dehydrogenase
LDH: lactate dehydrogenase
PADH: post-artesunate delayed haemolysis
PCR: polymerase chain reaction
RBC: red blood cell
PS: phosphatidyl-serine
KDIGO: Kidney Disease Improving Global Outcomes
NO: nitric oxide
